# Supplementary material for: An external focus of attention enhances table tennis backhand stroke accuracy in low-skilled players
Source: PLoS One. 2022 Dec 1;17(12):e0274717. doi: 10.1371/journal.pone.0274717 (PMC9714895; doi:10.1371/journal.pone.0274717)
Supplement: Attachment — Submitted filename: RESPONSE TO REVIEWERS COMMENTS.docx [file pone.0274717.s002.docx]

**RESPONSE TO REVIEWERS COMMENTS**

**Dear Editor and Reviewers,**

**The authors would like to thank the reviewers for their precious time and invaluable comments. We have carefully addressed all the comments. The corresponding changes and refinements made in the revised paper are summarized in our response below. We hope that the modifications and explanations will be acceptable to you.**

**REVIEWER #1**

1. Is the manuscript technically sound, and do the data support the conclusions?

The manuscript must describe a technically sound piece of scientific research with data that supports the conclusions. Experiments must have been conducted rigorously, with appropriate controls, replication, and sample sizes. The conclusions must be drawn appropriately based on the data presented.

Reviewer #1: Partly

**Response 1:** We agree with the reviewer and have made corrections.

Reviewer #2: Yes

**Response 1:** Thank you for your opinion

2. Has the statistical analysis been performed appropriately and rigorously?

Reviewer #1: N/A

**Response 2:** Thank you for your opinion

Reviewer #2: Yes

**Response 2:** Thank you for your opinion

3. Have the authors made all data underlying the findings in their manuscript fully available?

The [PLOS Data policy](http://www.plosone.org/static/policies.action#sharing) requires authors to make all data underlying the findings described in their manuscript fully available without restriction, with rare exception (please refer to the Data Availability Statement in the manuscript PDF file). The data should be provided as part of the manuscript or its supporting information, or deposited in a public repository. For example, in addition to summary statistics, the data points behind means, medians and variance measures should be available. If there are restrictions on publicly sharing data—e.g. participant privacy or use of data from a third party—those must be specified.

Reviewer #1: Yes

**Response 3:** Thank you for your opinion

Reviewer #2: Yes

**Response 3:** Thank you for your opinion

4. Is the manuscript presented in an intelligible fashion and written in standard English?

PLOS ONE does not copyedit accepted manuscripts, so the language in submitted articles must be clear, correct, and unambiguous. Any typographical or grammatical errors should be corrected at revision, so please note any specific errors here.

Reviewer #1: Yes

**Response 4:** Thank you for your opinion

Reviewer #2: Yes

**Response 4:** Thank you for your opinion

5. Review Comments to the Author

Reviewer #1: 1) The text needs to be improved grammatically.

**Response 5.1:** According to reviewers’ suggestions, manuscript has been reviewed and edited by a university native English speaker.

Introduction
2) At the beginning of the Introduction, you are needed to add a brief introduction of various teaching strategies in motor learning. Then you need to enter the attentional focus as an important strategy. And then, please focus on different types of attentional focus (i.e., internal vs. external).

**Response 5.2:** We agree with the reviewer and have made corrections.

3) Introduction is too weak. The most recent theory on focus of attention is the OTPIAML theory. The authors have not explained this theory in the Introduction. In addition, the novelty of this study is not clear. If using a racquet sport is enough for novelty of this experiment. What is the difference between racquet and not-racquet sports? These issues need to be explained more clearly in the Introduction.

**Response 5.3:** We agree with the reviewer and we change this part of the introduction.

4) Why you chose low-skilled players and not absolutely novices?

**Response 5.4:** Thank you for your question. We chose low-skilled players after 30 hours of table tennis classes because we wanted to verify how to conduct skill improvement.

5) How the students were low-skilled? Were they at the beginning of the semester, mid of semester, or have already finished their courses?

**Response 5.5:** Thank you for your question. The students were at the finished of the semester after 30 hours of table tennis classes.

6) Is the info about BMI and etc. important?

**Response 5.5:** Thank you for your question. BMI and etc. are very important in a research investigation, but this issue was not the purpose of our work.

7) The info about sample size should be placed in the Participants.

**Response 5.7:** We agree with the reviewer and we change this part of the methods.

8) The most important issue in your study is attentional focus instructions. However, it is not clear how these instructions were provided and applied. The procedure of this study has not been included. How the participant performed the strokes? If a person from opposite side throws the ball? Or the participant took the ball in his/her hand? Please explain the procedure in all detail.

**Response 5.8:** We respectfully disagree with the reviewer. The procedure of this study has been included. The experimental groups followed a similar experimental design, with one difference – instruction concerning focus of attention only in the practice phase (no attentional focus instructions were given in pre-test or post-test). Participants of G1 group (IF) were instructed to “concentrate on the hand holding the paddle”, G2 group (EFP) received instruction “concentrate on the ball”, and G3 group (EFD) – “concentrate on targets marked on the tennis table”. The experimenter gave attentional focus reminders at the beginning of each block. On each test day, the participants performed 15-minute warm-up routine (running and physical exercises). A 30-second break was provided after each block. The post-test was administered immediately after the practice session. No attentional focus instructions or reminders were given in retention test to assess the relatively permanent effects of the instructions. The experiment was conducted on consecutive days in a sports hall and was performed on a standard-size tennis table approved by International Table Tennis Federation (ANDRO Magnum SC, Germany). Each of the participants used the same professional paddle with the following characteristics: blade (ANDRO "Inizio ALL", Germany) and rubbers (DONIC "Liga", 2.0 mm, Germany). Plastic table tennis balls (DONIC "Coach ** P40+ Cell-Free", Germany) were delivered to the participants by a table tennis robot (NEWGY Robo-Pong 1050, DONIC, Newgy Industries Inc., Tennessee, USA) which was positioned in the middle of the table, on the opposite side of the participant. The robot was programmed as follows: ball speed (level 13), frequency – the time between balls served (1.5 s; 40 balls/min), rotation type (topspin), the angle of the robot head (level 6), the ball placement on the table (for backhand strokes – left and right position: level 3). The assessment developed by Poolton et al. [26] was used to determine the accuracy of the strokes during the pre-test, post-test and retention tests.

9) In relation to the comment “8”, it should be said that table tennis is considered a very fast sport because the acceleration of the ball is very high. My most concern is how the participants in G2 could concentrate on a ball which moves very fast. I am really confused about choosing this sport to examine proximal and distal effects. It is hard to believe that the participants could focus only on distal distance in table tennis, as table tennis players focus mostly on balls, not on the table. May shooting a basketball could provide more accurate findings.

**Response 9:** We agree with the reviewer that table tennis is difficult and complex sport. Therefore the robot was programmed as follows: ball speed (level 13), frequency – the time between of balls served (1.5 s; 40 balls/min), rotation type (topspin), the angle of the robot head (level 6), the ball placement on the table (for backhand strokes – left and right position: level 3).

Results
10) The table 1 looks very simple. Please use a different table design.

**Response 10:** We agree with the reviewer and we change this part of the results.

11) You talked about so many statistical tests; however, they were not reported in the Results?

**Response 11:** We agree with the reviewer and we change these parts.

Discussion
12) It was assumed that the discussion held heavily on the differences between proximal and distal effects in attentional focus. However, based on the Discussion, it is not fully understood why proximal and distal effects are the same in this study. Please clarify your findings.

**Response 12:** We agree with the reviewer and we change this part of the discussion.

13) Finally, an important reason for the effectiveness of external focus of attention is “goal-action coupling”. However, you have not pointed it out in your discussion. Please use it as well as the OPTIMAL theory for discussing your results.

**Response 13:** We agree with the reviewer and we change this part of the discussion.

Reviewer #2: This is an appropriate paper I have no hesitation in recommending it. The statistics used are appropriate and the conclusion is consistent and sound. While not entirely novel, it could deserve dissemination across the scientific community. I am looking forward to your future success.

**Response 5:** Thank you for your opinion
